# Identification and validation of early vital sign trajectory subphenotypes for risk stratification in sepsis-associated liver injury: a multicenter retrospective cohort study

**DOI:** 10.3389/fmed.2026.1835574

**Published:** 2026-10-09

**Authors:** Weihang Wang, Jing Ma, Bingqing Wang, Jiayang Wang, Zexi Zang, Qiyao Liu, Zhi Gao, Wei Chen, Yuxin Deng, Zhiqing Zhang, Yongfang Jiang

**Affiliations:** 1 Department of Infectious Diseases, The Second Xiangya Hospital, Central South University, Changsha, Hunan, China; 2 Department of Pathology, Affiliated Hospital of Nantong University, Nantong University, NanTong, Jiangsu, China; 3 Department of Pediatrics, The Alpert Medical School of Brown University, Women & Infants Hospital of Rhode Island, Providence, RI, United States

**Keywords:** dynamic subphenotypes, prognosis, risk stratification, sepsis-associated liver injury, vital sign trajectories

## Abstract

**Background:**

Sepsis-associated liver injury (SALI) is a serious clinical syndrome with heterogeneity in outcome among affected individuals. Physiological changes that are critical to outcome prediction are not considered in the existing system of stratification based on single-point observations. This could compromise the effectiveness of individualized treatment.

**Objective:**

To develop and validate dynamic clinical subphenotypes of SALI using early vital sign trajectories.

**Methods:**

This retrospective cohort study included 1,159 patients from MIMIC-IV as the derivation cohort and 168 patients from the Second Xiangya Hospital as the external validation cohort. Early vital sign trajectories within 12 h of ICU admission were summarized as trajectory-derived features and clustered using K-means. Candidate solutions (k = 2–6) were assessed using internal clustering metrics and bootstrap stability. A four-cluster solution was retained as an exploratory partition, considering trajectory interpretability and subgroup sizes. Clinical characteristics, organ support, and mortality outcomes were compared across the identified subphenotypes.

**Results:**

Four exploratory SALI subphenotypes with distinct early vital sign trajectories were identified, ranging from relatively stable profiles to marked hemodynamic and physiological stress. Subphenotype 3 exhibited the most stable vital signs and was associated with lower adjusted 28-day (HR 0.74, 95% CI 0.56–0.97) and 90-day mortality (HR 0.76, 95% CI 0.59–0.97) compared with the highest-risk Subphenotype 4. These prognostic differences were consistently replicated in the external cohort, supporting short- to intermediate-term prognostic generalizability.

**Conclusion:**

Early vital sign trajectories identified exploratory SALI groups associated with differences in 28-day and 90-day mortality. Their reproducibility, incremental prognostic value, and clinical utility require further evaluation.

## Introduction

Sepsis, as a life-threatening organ dysfunction caused by a dysregulated host response to infection, is a major cause of mortality worldwide and poses a significant burden on the health care system (1). This clinical condition is heterogeneous and varies widely in its immunological, physiological, and clinical manifestations, resulting in substantially different patient trajectories and responses to therapy (2, 3). Consequently, decades of clinical trials to find a universal therapy for sepsis have been unsuccessful, emphasizing the need to look beyond the “one-size-fits-all” approach and focus on the development of precision medicine approaches (2, 4). A major step forward in this direction has been the recognition of clinically relevant subphenotypes: distinct patient groups with similar underlying pathophysiology and prognosis (5).

Sepsis-associated liver injury (SALI) is a common and severe complication that occurs during the heterogeneous course of sepsis and reflects a state of systemic inflammation, hypoperfusion, and metabolic dysregulation (2). SALI has been independently associated with poorer patient outcomes; however, the clinical course varies significantly among patients. Current methods of patient stratification and outcome prediction in SALI are based on laboratory values and severity scores at a single time point (6). Sepsis is a dynamic process with physiological fluctuations occurring over a range of minutes to hours; however, the clinical course is currently assessed by a static measure, which might be inadequate to capture this important dynamic component of the sepsis process (2, 7). This limitation may mask underlying subphenotypes and hinder timely and individualized interventions.

To overcome this limitation, there has been a growing appreciation of the advantages of using dynamic trajectories from readily available data as a better approach for subphenotyping sepsis and other complex syndromes. The dynamic trajectory of vital signs and biomarkers has been demonstrated to reflect the real-time integration of complex host-pathogen interactions and inter-organ crosstalk (2, 8). Research has successfully used group-based trajectory modeling and other time-series clustering approaches to identify sepsis subphenotypes that have different outcomes and respond differentially to interventions such as the type of intravenous fluids used (5, 9, 10). For instance, the vital signs of septic patients during the initial hours of hospitalization have successfully identified four stable subphenotypes of sepsis that have different mortality rates and respond differentially to balanced crystalloids (1). Similarly, dynamic data such as temperature, inflammatory markers such as leukocytes, and metabolic markers such as lactate and anion gap have demonstrated good prognostic ability and reinforced the idea that evolution of these signs has more prognostic information than a snapshot of these signs at a single point in time (5, 11). This approach has also been effectively applied to other heterogeneous critical illnesses such as acute pancreatitis and has shown good promise (9, 12). Preliminary studies on SALI have effectively used this approach to begin to identify subphenotypes using a multi-algorithm approach and have demonstrated different pathophysiological profiles and different needs for personalized fluid resuscitation (13). However, a study of the early and dynamic physiological signs of SALI, such as vital signs, that distinguish subtypes of SALI patients at the time of Intensive Care Unit (ICU) admission is warranted (14, 15).

The current study seeks to address this gap by developing and validating dynamic clinical subphenotypes of critically ill patients with SALI, based on early vital sign trajectories. It is proposed that patients with SALI can be divided into distinct subphenotypes, with unique temporal trends in heart rate, blood pressure, respiratory rate, and temperature in the first 12 h of ICU admission. Furthermore, it is proposed that these subphenotypes will show marked differences in baseline characteristics, biomarker levels, and short- and long-term mortality outcomes. To validate these hypotheses, the current study will use a retrospective multicenter cohort study design using the Medical Information Mart for Intensive Care IV (MIMIC-IV, version 3.1) database for subphenotype development and an independent cohort for validation of the association with ICU, 28-day, 90-day, and 365-day mortality outcomes. The aims of the current study are: (1) to identify data-driven subphenotypes of SALI using feature-based clustering of early vital sign trajectories; (2) to characterize the subphenotypes by comparing demographic features, laboratory measurements, and organ support requirements across groups; and (3) to evaluate the association of these trajectory-derived groups with ICU, 28-day, 90-day, and 365-day mortality outcomes. By combining trajectory-informed feature extraction with outcome assessment, this study seeks to provide a dynamic framework for early risk stratification in SALI while recognizing that any identified subphenotypes require further validation.

## Materials and methods

### Study design and population

To develop dynamic clinical subphenotypes and explore the unique clinical traits across subphenotypes to guide precision risk stratification, we conducted a retrospective, multicenter observational cohort study. SALI was defined as new-onset liver injury occurring within 48 h after sepsis onset, characterized by at least one of the following abnormalities: (1) total bilirubin ≥2 mg/dL, or (2) alanine aminotransferase (ALT) or aspartate aminotransferase (AST) levels >2 × the upper limit of normal (40 U/L for both). Eligibility for the study population encompassed adult ICU patients with sepsis who subsequently developed SALI. In cases of multiple ICU admissions, only the first ICU admission was included for analysis. Exclusion criteria included age <18 years, ICU length of stay shorter than 48 h, death within 48 h after ICU admission, chronic liver disease and liver injury occurring before sepsis onset, and insufficient early vital sign data, defined as fewer than three observation time points within the first 12 h after ICU admission. We used an extensive repository of electronic health record (EHR) data extracted from two real-world critical care databases. These databases included the Medical Information Mart for Intensive Care IV (MIMIC-IV) database, which contains detailed electronic health record data from critically ill patients admitted to Beth Israel Deaconess Medical Center and the Second Xiangya Hospital of Central South University (XY2), a comprehensive multicenter intensive care hospital. After data preprocessing, the MIMIC-IV dataset was designated as the development cohort for sepsis induced liver injury subphenotyping, and XY2 datasets served as the external validation cohort.

## Model development for subphenotyping

Vital sign trajectories data preparation: Vital signs, including heart rate, respiratory rate, body temperature, systolic blood pressure (SBP), and diastolic blood pressure (DBP), were extracted during the first 12 h after ICU admission. Measurements were aggregated on an hourly basis, and when multiple measurements were available within the same hour, their mean values were calculated. To construct complete trajectories, patients with fewer than three available vital sign measurements within the first 12 h were excluded to ensure that trajectory estimation was based on sufficient longitudinal information rather than imputed values. For the remaining patients, linear interpolation was applied at the individual level when at least two valid observations were available for a given vital sign; if only one observation was available, forward and backward filling was performed. Missing values after interpolation were imputed using cohort-level mean values. The resulting hourly vital sign trajectories were subsequently transformed into a wide-format representation for feature extraction.

An unsupervised clustering framework was employed to identify subphenotypes based on early vital sign trajectories. For each patient, trajectory-derived features were extracted from the first 12 h of vital sign data, including quadratic polynomial coefficients, mean values, standard deviations, and overall temporal trends for each vital sign. These features were standardized and entered into a K-means clustering model. Candidate clustering solutions (k = 2–6) were evaluated using the silhouette score, Calinski–Harabasz index, Davies–Bouldin index, gap statistic, bootstrap stability, and subgroup sizes (supplementary Table 1). These criteria did not identify a single optimal solution, and k = 4 was not the most stable partition. The four-cluster solution was retained for exploratory characterization based on trajectory interpretability and subgroup sizes, rather than as a statistically established optimal solution. Model application and external assignment: For external application, each patient's feature vector was standardized using the scaler derived from the development cohort. Patients were then assigned to the nearest cluster centroid based on Euclidean distance in the standardized feature space.

## Clinical characteristics and outcomes between subphenotypes

Clinical characteristics including demographic data (age and sex), baseline laboratory measurements, organ support therapies, and severity scores were collected. Continuous variables were summarized as mean ± standard deviation, and categorical variables were presented as counts (percentages). Differences between subphenotypes were assessed using Kruskal–Wallis tests for continuous variables and *χ*^2^ tests for categorical variables. ICU, 28-day, and 90-day mortality were assessed in both cohorts, whereas 365-day mortality was assessed only in the development cohort. In the external cohort, reliable mortality follow-up was available only through day 90. Patients without a recorded death were censored at the earlier of their last known follow-up date or day 90. Survival differences across subphenotypes were evaluated using Kaplan–Meier curves and log-rank tests, followed by Cox proportional hazards regression models adjusted for age, sex, sofa score and vasopressor use. In the ICU survival analysis, ICU mortality was designated as the primary event, ICU length of stay was defined as the time scale, and patients discharged alive were censored at their discharge time. Consequently, the derived Kaplan–Meier curve reflects cumulative survival probabilities under censoring, rather than representing a crude ICU mortality rate.

## Missing data management

For vital sign trajectories, missing values were handled using patient-level linear interpolation and mean imputation, as described above. For baseline clinical variables used in clustering and prediction models, missing values were imputed using median imputation. Variables with excessive missingness were excluded from downstream analyses. The implausible BMI value was treated as missing and imputed using the median BMI of the development cohort. The patient was retained in the study, and the development cohort therefore remained at 1,159 patients.

## Statistical analysis

All analyses were conducted using Python (version 3.12). Statistical analyses were performed using SciPy and lifelines packages, and machine learning models were implemented using scikit-learn. All tests were two-sided, and a *P* value <0.05 was considered statistically significant. Comparisons of baseline clinical characteristics were conducted primarily for descriptive and exploratory purposes rather than for confirmatory hypothesis testing. Therefore, no formal adjustment for multiple testing was applied. Consequently, some statistically significant findings may be attributable to type I error and should be interpreted with caution.

## Results

### Cohort derivation and baseline characteristics

In total, 7,234 adult ICU patients with sepsis were identified from the development (MIMIC-IV) and external validation (XY2) database (Figure 1). After filtering and screening, 1,159 patients from MIMIC-IV constituted the derivation cohort, and 168 patients from the XY2 database constituted the external validation cohort. The main reasons for exclusion from this study were cases with liver injuries before sepsis or lack of liver injuries within 48 h. The mean age of patients in the derivation cohort was 65.44 ± 16.82 years, and 60.4% were male. The mean Sequential Organ Failure Assessment (SOFA) and Acute Physiology and Chronic Health Evaluation II (APACHE II) scores on admission to ICU were 8.11 ± 3.77 and 25.73 ± 8.23, respectively (Figures 2A,B, Table 1). These scores indicated a patient population with moderate to severe illness.

**Figure 1:**
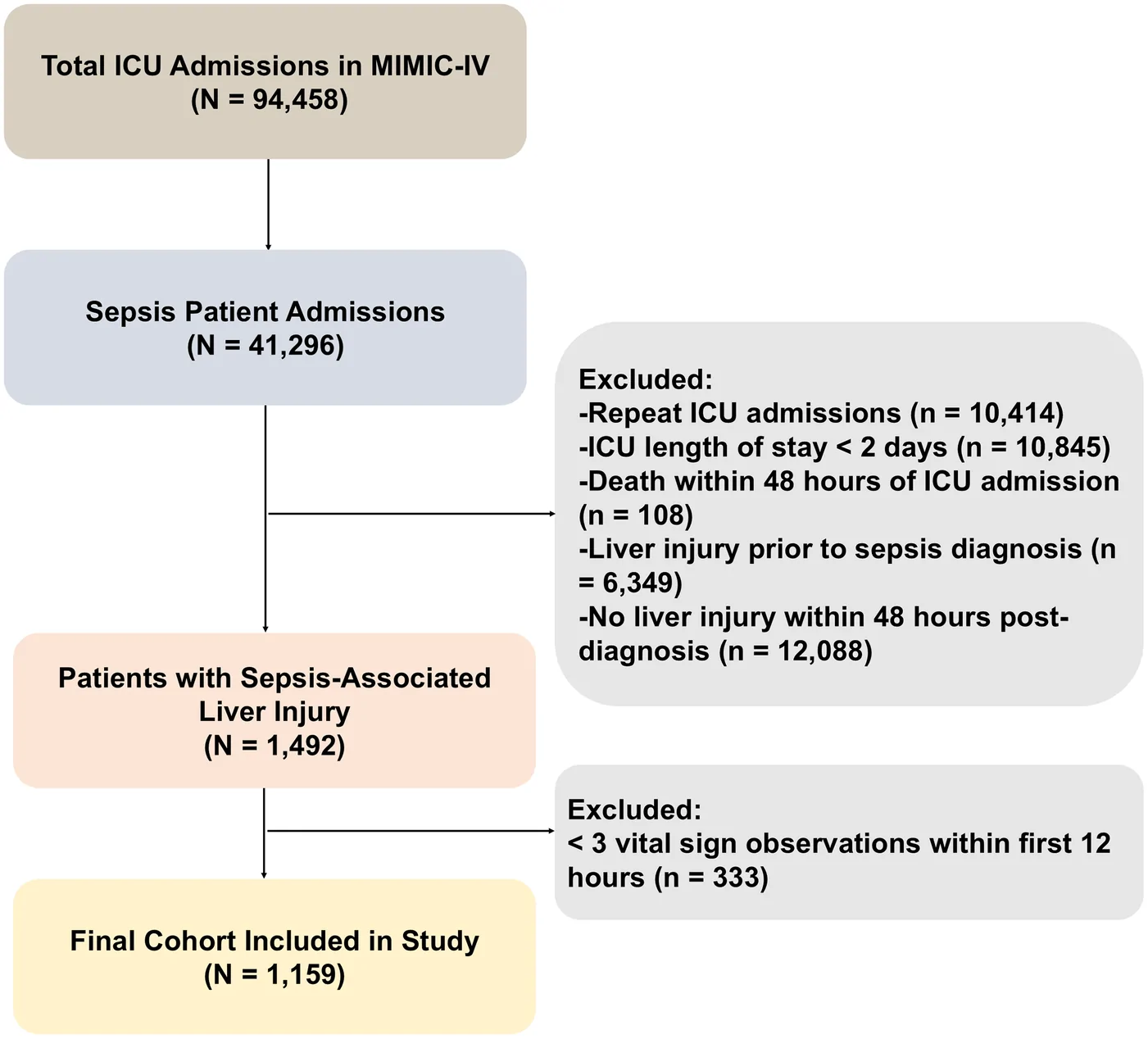
Flow diagram of patient selection for the MIMIC-IV development cohort.

**Figure 2:**
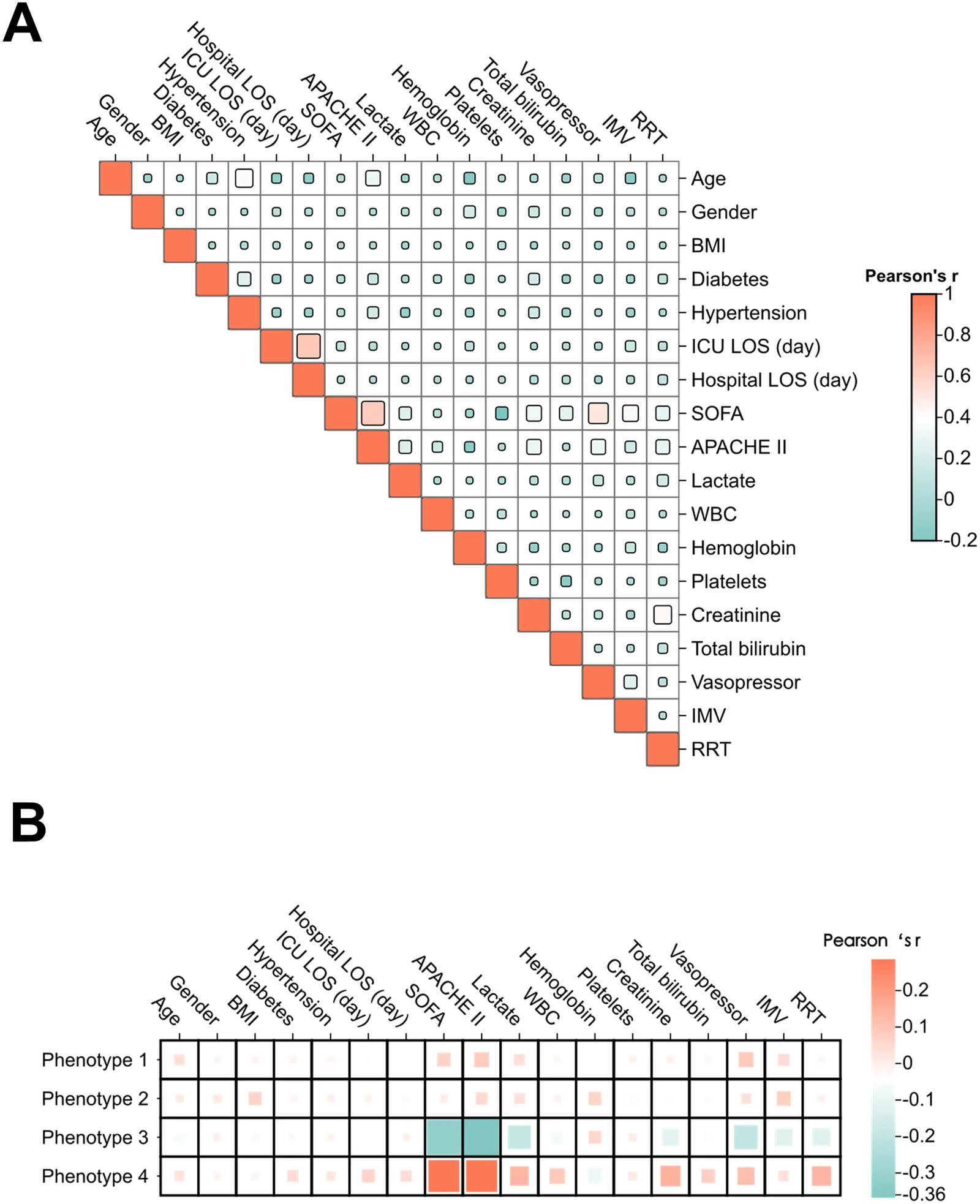
**(A)** displays Pearson correlations between variables. **(B)** displays Pearson correlation between variables and subphenotypes.

**Table 1:** The clinical characteristics of subphenotypes in development cohort.

Characteristic	Overall	Phenotype 1	Phenotype 2	Phenotype 3	Phenotype 4	*p*-value
*N*	1,159	137	180	469	373	
Age, years	65.44 ± 16.82	67.17 ± 17.81	65.79 ± 17.98	64.18 ± 16.72	66.20 ± 15.93	0.108
Gender, *n* (%)						0.904
Male	700 (60.4%)	80 (58.4%)	112 (62.2%)	285 (60.8%)	223 (59.8%)	
Female	459 (39.6%)	57 (41.6%)	68 (37.8%)	184 (39.2%)	150 (40.2%)	
BMI, kg/m²	29.79 ± 8.69	29.49 ± 8.94	29.32 ± 7.80	29.54 ± 9.05	30.42 ± 8.56	0.204
Diabetes, *n* (%)	380 (32.8%)	43 (31.4%)	56 (31.1%)	147 (31.3%)	134 (35.9%)	0.482
Hypertension, *n* (%)	731 (63.1%)	82 (59.9%)	114 (63.3%)	294 (62.7%)	241 (64.6%)	0.796
ICU length of stay (day)	9.02 ± 8.34	8.20 ± 7.05	8.99 ± 7.99	8.63 ± 8.01	9.84 ± 9.27	0.140
Hospital length of stay (day)	17.66 ± 14.86	15.91 ± 11.21	16.72 ± 14.18	17.74 ± 14.61	18.66 ± 16.56	0.771
SOFA	8.11 ± 3.77	8.85 ± 3.71	8.20 ± 3.62	6.61 ± 3.39	9.67 ± 3.60	<0.001
APACHE II	25.73 ± 8.23	27.69 ± 8.09	26.75 ± 8.61	22.06 ± 7.31	29.13 ± 7.32	<0.001
Lactate, mmol/L	3.00 ± 2.51	3.30 ± 2.98	3.24 ± 2.71	2.24 ± 1.51	3.55 ± 2.83	<0.001
WBC, ×10⁹/L	14.88 ± 16.53	13.94 ± 9.62	14.60 ± 15.12	13.48 ± 7.90	17.14 ± 24.92	0.069
Hemoglobin, g/dL	10.93 ± 2.67	10.64 ± 2.57	11.34 ± 2.42	11.13 ± 2.52	10.59 ± 2.95	0.001
Platelets, ×10⁹/L	212.60 ± 128.19	211.44 ± 122.49	203.49 ± 106.01	213.99 ± 113.75	215.68 ± 154.73	0.557
Creatinine, mg/dL	1.84 ± 1.68	1.82 ± 1.75	1.70 ± 1.28	1.60 ± 1.40	2.21 ± 2.04	<0.001
Total bilirubin, mg/dL	1.36 ± 2.13	1.20 ± 1.21	1.19 ± 1.34	1.27 ± 1.86	1.60 ± 2.82	0.044
Vasopressor use, *n* (%)	715 (61.7%)	101 (73.7%)	118 (65.6%)	235 (50.1%)	261 (70.0%)	<0.001
IMV, *n* (%)	845 (72.9%)	108 (78.8%)	146 (81.1%)	311 (66.3%)	280 (75.1%)	<0.001
RRT, *n* (%)	82 (7.1%)	8 (5.8%)	13 (7.2%)	15 (3.2%)	46 (12.3%)	<0.001

### Identification of early vital sign trajectory subphenotypes

Using unsupervised K-means clustering on the trajectory feature set from the first 12 h of ICU admission, four distinct subphenotypes were identified within the derivation cohort (Figures 3A–E). These subphenotypes showed distinct early physiological patterns. Subphenotype 1 had the lowest initial blood pressure, followed by an early rise in both systolic and diastolic pressure toward hour 6–8, together with transient increases in heart rate and respiratory rate. Subphenotype 2 showed the highest initial systolic and diastolic blood pressure, with a pronounced early decline toward intermediate levels, while heart rate and respiratory rate remained only moderately elevated and relatively stable. Subphenotype 3 showed comparatively near-normal and stable vital signs, with only mild changes over time. Subphenotype 4 had the highest initial heart rate and respiratory rate and maintained a relatively stressed cardiopulmonary profile, but its blood pressure remained in a moderately low-to-intermediate range rather than representing the lowest-pressure group throughout the observation window.

**Figure 3:**
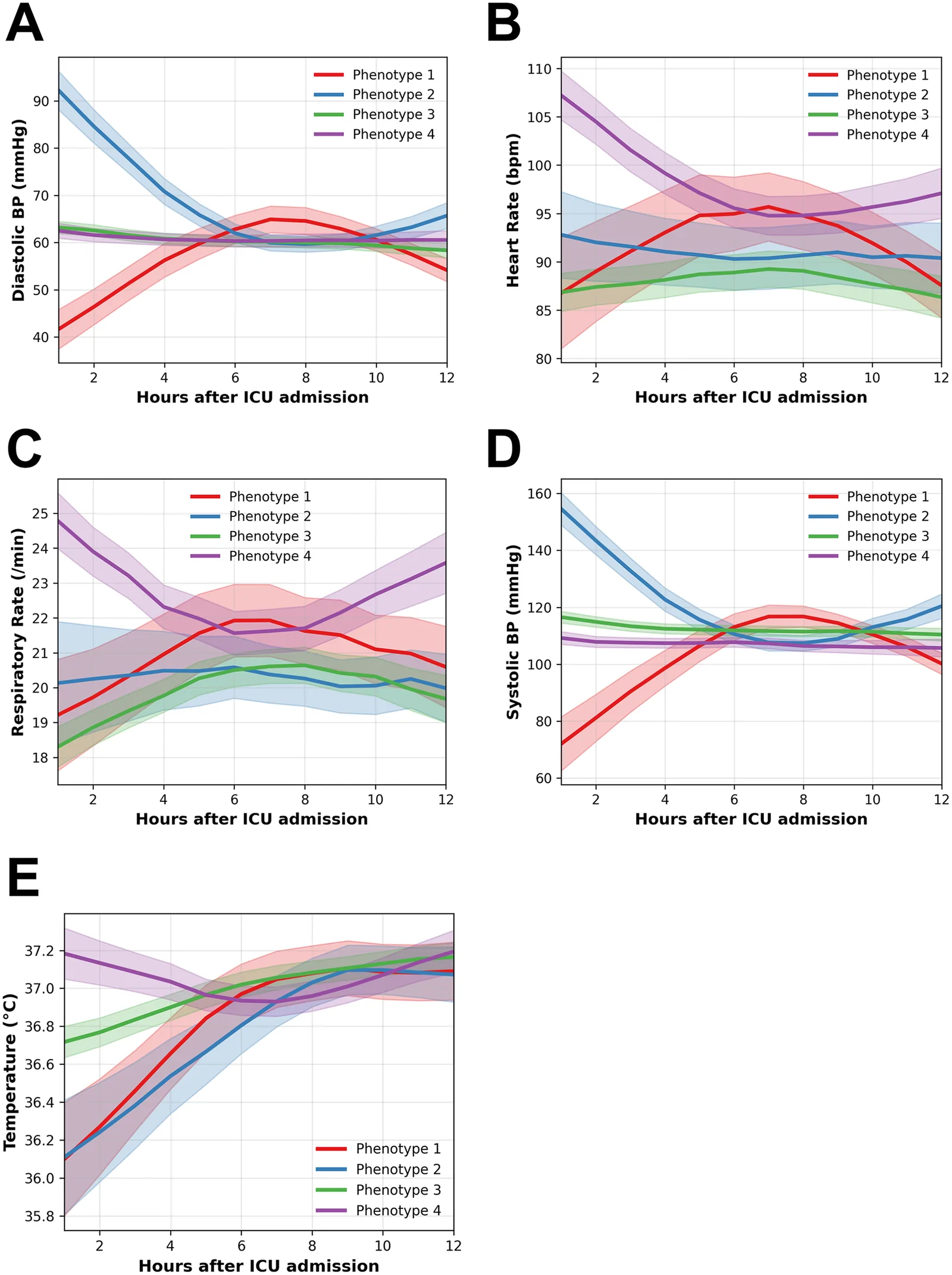
Early vital sign trajectories across the four trajectory-derived subphenotypes in the development cohort. Lines represent mean values and shaded areas indicate 95% confidence intervals. **(A)** Diastolic blood pressure, **(B)** heart rate, **(C)** respiratory rate, **(D)** systolic blood pressure, and **(E)** body temperature. The four groups were derived from K-means clustering of standardized trajectory features extracted from the first 12 h after ICU admission.

### Clinical and biological characterization of subphenotypes

The clinical and biological characteristics of the four subphenotypes in the development cohort are presented in Table 1, and those in the validation cohort are shown in Supplementary Table 2. For development cohort, there were no differences in the age, gender, and BMI of the subphenotypes. However, the subphenotypes showed differences in illness severity and biological characteristics (Figure 2B). Subphenotype 4 showed the highest illness severity in the development cohort, with higher SOFA and APACHE II scores than Subphenotype 1 (SOFA: 9.67 ± 3.60 vs. 8.85 ± 3.71; APACHE II: 29.13 ± 7.32 vs. 27.69 ± 8.09; both *p* < 0.001). Admission lactate levels were lowest in Subphenotype 3 and highest in Subphenotype 4, while Subphenotypes 1 and 2 showed intermediate values (*p* < 0.001). Subphenotype 4 had the highest level of total bilirubin (1.60 ± 2.82 mg/dL compared to others, *p* = 0.044). Organ support use varied across subphenotypes. Vasopressor use was highest in Subphenotype 1 (73.7%), followed by Subphenotype 4 (70.0%). Invasive mechanical ventilation was most frequent in Subphenotype 2 (81.1%), whereas RRT was most frequent in Subphenotype 4 (12.3%). Subphenotype 3 had the lowest use of all three therapies (overall between-group *p* < 0.001 for each). Supplementary Table 3 compares the baseline characteristics between the development cohort (*n* = 1,159) and the validation cohort (*n* = 168). Significant differences were observed between the development and validation cohorts in several baseline characteristics, including age, BUN, ALT, potassium, hemoglobin, sex, comorbidities such as renal disease and cancer, and vasopressor use (Supplementary Table 3), indicating substantial clinical heterogeneity between the two cohorts.

### Differences in outcome for subphenotypes

Survival analysis showed significant prognostic differences in patient outcomes for these subphenotypes. Although mortality in the ICU did not reach statistical significance (log-rank *p* = 0.0786) (Figure 4A), differences in 28-day, 90-day, and 365-day mortality showed distinct separation in Kaplan–Meier survival curves (log-rank *p* < 0.001 for all) (Figures 4B–D). Subphenotype 3 had the best survival outcomes for all time points, while Subphenotype 4 had the worst outcomes.

**Figure 4:**
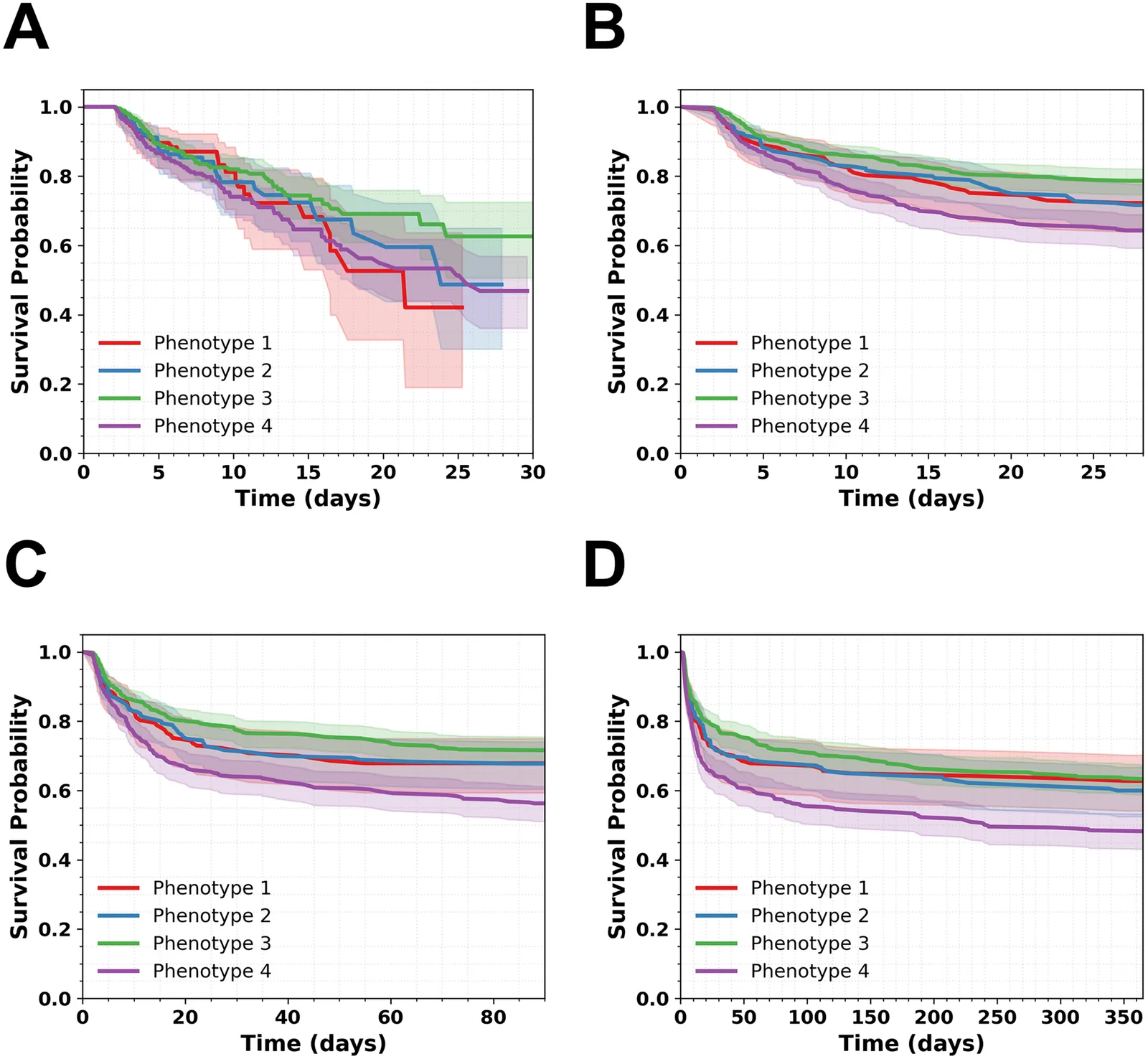
Kaplan–Meier survival curves by subphenotype in the development cohort. **(A)** ICU survival, with ICU death treated as the event and live ICU discharge treated as censoring (log-rank *p* = 0.0786). **(B)** 28-day survival (log-rank *p* = 0.0001). **(C)** 90-day survival (log-rank *p* = 0.0000). **(D)** 365-day survival (log-rank *p* = 0.0000).

These results were confirmed using multivariable Cox proportional hazards models. Model 1 was adjusted for age and sex, and Model 2 was additionally adjusted for SOFA score and vasopressor use, as presented in Tables 2–5. Using Subphenotype 4 as the reference group (highest risk), Subphenotype 3 was associated with a significantly lower risk of mortality across multiple time points in the development cohort after adjustment for age, sex, SOFA score, and vasopressor use (Adjusted Model 2). Specifically, Subphenotype 3 showed reduced hazards for 28-day mortality (adjusted HR 0.74, 95% CI 0.56–0.97, *p* = 0.031), 90-day mortality (adjusted HR 0.76, 95% CI 0.59–0.97, *p* = 0.028), and a borderline significant reduction for 365-day mortality (adjusted HR 0.80, 95% CI 0.64–1.00, *p* = 0.050). However, no significant association was observed for ICU mortality after full adjustment (adjusted HR 0.82, 95% CI 0.59–1.14, *p* = 0.235). In contrast, Subphenotypes 1 and 2 did not show statistically significant differences compared with Subphenotype 4 for ICU, 28-day, or 90-day mortality in the fully adjusted model. Nevertheless, both Subphenotype 1 and Subphenotype 2 retained significant associations with lower 365-day mortality, with adjusted hazard ratios of 0.69 (95% CI 0.50–0.94, *p* = 0.017) and 0.75 (95% CI 0.57–0.99, *p* = 0.042), respectively. Overall, the initially observed protective effects of Subphenotypes 1 and 2 in crude and partially adjusted models were substantially attenuated after further adjustment for disease severity and treatment-related variables, suggesting that baseline clinical severity may partly explain these associations.

**Table 2:** Multivariate cox regression result between subphenotypes of ICU mortality in development and validation cohorts.

Model	Phenotype 4	Phenotype 1		Phenotype 2		Phenotype 3	
		HR (95%CI)	*P*-value	HR (95%CI)	*P*-value	HR (95%CI)	*P*-value
Development cohort (MIMIC-IV)							
ICU mortality							
Crude	Reference	0.855 (0.554, 1.318)	0.478	0.793 (0.539, 1.166)	0.238	0.672 (0.496, 0.910)	0.010
Adjusted1^a^	Reference	0.815 (0.528, 1.257)	0.355	0.769 (0.522, 1.131)	0.182	0.681 (0.503, 0.922)	0.013
Adjusted2^a^	Reference	0.891 (0.575, 1.380)	0.605	0.827 (0.561, 1.221)	0.340	0.820 (0.592, 1.137)	0.235
External validation cohort (XY2)							
ICU mortality							
Crude	Reference	0.800 (0.223, 2.869)	0.732	0.552 (0.154, 1.978)	0.361	0.956 (0.428, 2.133)	0.912
Adjusted1	Reference	0.666 (0.177, 2.508)	0.548	0.563 (0.155, 2.040)	0.382	0.857 (0.380, 1.932)	0.709
Adjusted2	Reference	0.703 (0.186, 2.659)	0.603	0.561 (0.155, 2.028)	0.378	0.872 (0.387, 1.965)	0.742

a Adjusted Model 1: age and sex. Adjusted Model 2 (development cohort): age, sex, SOFA score, and vasopressor use. Adjusted Model 2 (external validation cohort): age, sex, and vasopressor use.

**Table 3:** Multivariate cox regression result between subphenotypes of 28-day mortality in development and validation cohorts.

Model	Phenotype 4	Phenotype 1		Phenotype 2		Phenotype 3	
		HR (95%CI)	*P*-value	HR (95%CI)	*P*-value	HR (95%CI)	*P*-value
Development cohort (MIMIC-IV)							
28-day mortality							
Crude	Reference	0.737 (0.514, 1.057)	0.097	0.748 (0.541, 1.033)	0.077	0.545 (0.421, 0.707)	＜0.001
Adjusted1^a^	Reference	0.699 (0.487, 1.003)	0.052	0.720 (0.521, 0.995)	0.046	0.566 (0.436, 0.734)	＜0.001
Adjusted2^a^	Reference	0.794 (0.552, 1.143)	0.215	0.842 (0.607, 1.169)	0.305	0.735 (0.557, 0.972)	0.031
External validation cohort (XY2)							
28-day mortality							
Crude	Reference	0.463 (0.193, 1.108)	0.084	0.387 (0.171, 0.878)	0.023	0.396 (0.222, 0.707)	0.002
Adjusted1	Reference	0.337 (0.135, 0.843)	0.02	0.359 (0.157, 0.822)	0.015	0.363 (0.202, 0.650)	0.001
Adjusted2	Reference	0.343 (0.137, 0.860)	0.023	0.363 (0.159, 0.830)	0.016	0.367 (0.205, 0.658)	<0.001

* Adjusted Model 1: age and sex. Adjusted Model 2 (development cohort): age, sex, SOFA score, and vasopressor use. Adjusted Model 2 (external validation cohort): age, sex, and vasopressor use.

**Table 4:** Multivariate cox regression result between subphenotypes of 90-day mortality in development and validation cohorts.

Model	Phenotype 4	Phenotype 1		Phenotype 2		Phenotype 3	
		HR (95%CI)	*P*-value	HR (95%CI)	*P*-value	HR (95%CI)	*P*-value
Development cohort (MIMIC-IV)							
90-day mortality							
Crude	Reference	0.686 (0.492, 0.958)	0.027	0.684 (0.507, 0.923)	0.013	0.579 (0.460, 0.728)	＜0.001
Adjusted1^a^	Reference	0.652 (0.467, 0.910)	0.012	0.654 (0.485, 0.883)	0.006	0.600 (0.477, 0.755)	＜0.001
Adjusted2^a^	Reference	0.733 (0.524, 1.026)	0.070	0.752 (0.555, 1.020)	0.067	0.758 (0.593, 0.970)	0.028
External validation cohort (XY2)							
90-day mortality							
Crude	Reference	0.744 (0.377, 1.466)	0.392	0.560 (0.290, 1.080)	0.084	0.436 (0.259, 0.735)	0.002
Adjusted1	Reference	0.544 (0.267, 1.108)	0.093	0.528 (0.272, 1.026)	0.059	0.394 (0.233, 0.666)	0.001
Adjusted2	Reference	0.552 (0.271, 1.126)	0.102	0.531 (0.273, 1.031)	0.061	0.395 (0.233, 0.669)	<0.001

a Adjusted Model 1: age and sex. Adjusted Model 2 (development cohort): age, sex, SOFA score, and vasopressor use. Adjusted Model 2 (external validation cohort): age, sex, and vasopressor use.

**Table 5:** Cox regression analyses of 365-day mortality in the development cohort.

Model	Phenotype 4	Phenotype 1		Phenotype 2		Phenotype 3	
		HR (95%CI)	*P*-value	HR (95%CI)	*P*-value	HR (95%CI)	*P*-value
Development cohort (MIMIC-IV)							
365-day mortality							
Crude	Reference	0.654 (0.480, 0.891)	0.007	0.702 (0.535, 0.920)	0.010	0.617 (0.502, 0.758)	W
Adjusted1^a^	Reference	0.617 (0.453, 0.841)	0.002	0.661 (0.504, 0.867)	0.003	0.641 (0.521, 0.787)	<0.001
Adjusted2^a^	Reference	0.685 (0.502, 0.936)	0.017	0.752 (0.571, 0.989)	0.042	0.802 (0.643, 1.000)	0.050

a Adjusted Model 1: age and sex. Adjusted Model 2 (development cohort): age, sex, SOFA score, and vasopressor use. Adjusted Model 2 (external validation cohort): age, sex, and vasopressor use.

### External validation

In the external cohort, the overall prognostic ranking across subphenotypes was directionally similar for 28-day and 90-day mortality. Because reliable mortality follow-up in the external cohort was limited to 90 days, external validation was restricted to ICU, 28-day, and 90-day mortality. Outcomes beyond day 90 could not be reliably ascertained. As shown in Tables 2–4, the risk stratification trend was largely consistent with the results obtained in the development cohort. Because SOFA score, APACHE II score, and lactate were unavailable in this cohort, and vasopressor use was recorded in only 5 of 168 patients, severity adjustment in the external analyses was limited. Compared with Subphenotype 4, Subphenotype 3 was not significantly associated with ICU mortality after adjustment (HR 0.87, 95% CI 0.39–1.97, *p* = 0.74). However, Subphenotype 3 remained significantly associated with lower 28-day mortality (HR 0.37, 95% CI 0.21–0.66, *p* < 0.001), and 90-day mortality (HR 0.40, 95% CI 0.23–0.67, *p* < 0.001).

## Discussion

This multicenter cohort study identified four exploratory trajectory-derived subphenotypes of sepsis-associated liver injury based on early vital sign patterns during the first 12 h of ICU admission. Importantly, internal clustering metrics did not converge on a single optimal k value, and the global cluster separation for the 4-cluster solution was modest. Accordingly, the retained 4-cluster partition should be interpreted as a pragmatic and clinically oriented grouping structure rather than as a definitive latent taxonomy of SALI. Within that framework, the identified groups still showed clinically relevant heterogeneity in illness severity, organ support requirements, and mortality patterns, with Subphenotypes 1 and 4 being the most severe in terms of unstable hemodynamics, elevated lactate and creatinine levels, and vasopressor and mechanical ventilatory requirements (Table 1). The subphenotypes also emerged as a significant predictor of mortality in a multivariate analysis, patients in Subphenotype 3, characterized by stable or near-normal vital signs, had the best survival outcomes, while patients in Subphenotype 4, characterized by severe and unstable vital signs, had the worst survival outcomes at all time points, i.e., 28, 90, and 365 days, respectively (Tables 3–5, Figure 4). These findings suggest that early vital sign trajectories may capture prognostically relevant heterogeneity in SALI. However, the exploratory clustering results do not establish discrete biological subtypes or demonstrate added prognostic value beyond single-time-point assessments.

These findings support and extend the growing body of literature advocating precision medicine approaches for sepsis and its complications. Previous studies have demonstrated that dynamic physiological information provides greater prognostic insight than static measurements. For example, Bhavani et al. identified sepsis subphenotypes based on 8-hour vital-sign trajectories that were associated with differential outcomes and treatment responses, while Zhang et al. showed that dynamic SOFA trajectories outperform static SOFA measurement for prognostic assessment (2, 16). Consistent with these observations, our findings demonstrate that early physiological trajectories capture clinically meaningful heterogeneity within patients with SALI. Unlike previous prediction models based primarily on admission variables, our framework focuses on the dynamic evolution of physiological instability during the first 12 h of ICU admission. Li et al. developed a nomogram for predicting SALI using admission characteristics (17), whereas our study further stratifies prognosis among patients with established SALI according to early vital-sign trajectories. Compared with the 72-hour SOFA trajectory model proposed by Xu et al. (16), our approach uses a substantially shorter observation window and higher-frequency physiological measurements, potentially enabling earlier identification of high-risk patients. Furthermore, while Miao et al. identified four SALI subphenotypes using baseline clinical and laboratory variables (3), our trajectory-based framework characterizes patients according to their dynamic physiological evolution rather than static composite scores, thereby providing complementary information on disease progression.

The subphenotypes largely reflected differences in illness severity. Subphenotype 3 had the lowest severity scores and organ support use, whereas Subphenotype 4 had the highest severity scores and RRT use. Vasopressor use and invasive mechanical ventilation were highest in Subphenotypes 1 and 2, respectively. After adjustment, only Subphenotype 3 remained associated with lower 28-day and 90-day mortality compared with Subphenotype 4. These findings suggest that the groups primarily represent differences in early physiological severity; whether they reflect distinct biological mechanisms requires further investigation.

This study's emphasis on the very initial phase (≤48 h) of SALI is a significant contribution. This is consistent with the clinical knowledge that a severe form of SALI, hypoxic hepatitis, usually appears during this window (18). Our framework offers a risk assessment tool by modeling data from the first 12 h of ICU admission. A balanced assessment of this study calls for awareness of its strengths and limitations. Importantly, the attenuation of the associations for Subphenotypes 1 and 2 after adjustment indicates that trajectory-based subphenotypes complement rather than replace conventional severity scores. While static scores quantify illness severity at a single time point, trajectory-based classification captures the dynamic evolution of physiological responses and may therefore provide additional insight into patient heterogeneity. The observed association with 365-day mortality should be interpreted cautiously and requires further prospective validation.

The identified trajectory-based subphenotypes may provide a complementary approach for early risk stratification following initial ICU stabilization. However, whether trajectory-based classification improves clinical decision-making beyond existing risk assessment tools remains to be determined. Because subphenotype assignment was based on the first 12 h of physiological data, the present framework should be regarded as an early post-admission risk stratification approach rather than a tool for immediate decision-making at ICU admission. Future studies should evaluate whether comparable prognostic performance can be achieved using shorter observation windows (e.g., 6 or 8 h). In addition, successful implementation will require automated data preprocessing, integration with electronic health record systems, and prospective workflow validation before routine clinical application (19, 20).

Several limitations should be acknowledged. First, differences in patient characteristics, illness severity, and clinical practice between cohorts may limit generalizability, requiring larger and geographically diverse external validations. Second, subphenotype assignment may be sensitive to missing-data handling and clustering choices. The four-cluster solution showed modest separation and limited bootstrap stability (ARI, 0.47 ± 0.18), with a lower mean ARI than the two-cluster (0.78) and three-cluster (0.53) solutions. These findings support treating the groups as exploratory trajectory-derived strata rather than established biological subtypes. Third, partial convergence of trajectories by hours 6–8 may reflect treatment effects, physiological stabilization, or regression to the mean, rather than distinct causal mechanisms. Fourth, residual confounding remains possible because SOFA, APACHE II, and lactate were not uniformly available across cohorts. Fifth, reliable mortality follow-up in the external cohort was limited to 90 days, preventing external validation of longer-term associations. Finally, prospective multicenter studies are needed to establish the clinical utility of trajectory-based phenotyping.

In conclusion, this study identified four exploratory groups based on early vital sign trajectories in patients with SALI. Subphenotype 3 was associated with lower adjusted 28-day and 90-day mortality than Subphenotype 4 in both cohorts. However, modest cluster stability, limited severity adjustment in the external cohort, and the absence of reliable external follow-up beyond 90 days constrain interpretation. Further studies are needed to assess reproducibility and incremental prognostic value before clinical application

## Data Availability

The raw data supporting the conclusions of this article will be made available by the authors, without undue reservation.
