# Supplementary material for: Identification and validation of early vital sign trajectory subphenotypes for risk stratification in sepsis-associated liver injury: a multicenter retrospective cohort study
Source: Front Med (Lausanne). 2026 Oct 9;13:1835574. doi: 10.3389/fmed.2026.1835574 (PMC13690518; doi:10.3389/fmed.2026.1835574)
Supplement: Supplementary file 1 [file Datasheet1.docx]

**Supplementary Table S1. Internal evaluation of candidate clustering solutions (k = 2–6), including silhouette score, Calinski-Harabasz index, Davies-Bouldin index, gap statistic, bootstrap stability, and subgroup size distribution.**

| **k** | **Silhouettescore** | **Calinski Harabasz**  **index** | **Davies Bouldin index** | **Gap statistic** | **ARI**  **mean** | **ARI SD** | **Cluster sizes** |
| --- | --- | --- | --- | --- | --- | --- | --- |
| 2 | 0.11 | 160.44 | 2.38 | 2.59 | 0.78 | 0.15 | 1: 634, 2: 525 |
| 3 | 0.12 | 131.06 | 2.34 | 2.62 | 0.53 | 0.25 | 1: 628, 2: 222, 3: 309 |
| 4 | 0.08 | 116.72 | 2.26 | 2.64 | 0.47 | 0.18 | 1: 137, 2: 180, 3: 469, 4: 373 |
| 5 | 0.07 | 107.58 | 2.1 | 2.66 | 0.48 | 0.13 | 1: 408, 2: 338, 3: 141, 4: 175, 5: 97 |
| 6 | 0.08 | 99.89 | 2.03 | 2.68 | 0.44 | 0.11 | 1: 218, 2: 232, 3: 130, 4: 382, 5: 88, 6: 109 |

**Supplementary Table 2. The clinical characteristics of subphenotypes in validation cohort**

| **Variable** | **Overall** | **Phenotype 1** | **Phenotype 2** | **Phenotype 3** | **Phenotype 4** | **p-value** |
| --- | --- | --- | --- | --- | --- | --- |
| **N** | 168 | 19 | 26 | 68 | 55 |  |
| **Age, years** | 63.23±14.84 | 73.47±11.84 | 61.00±15.23 | 64.88±12.49 | 58.71±16.42 | 0.001 |
| **Gender, n (%)** |  |  |  |  |  | 0.276 |
| Male | 125 (74.4%) | 12 (63.2%) | 17 (65.4%) | 55 (80.9%) | 41 (74.5%) |  |
| Female | 43 (25.6%) | 7 (36.8%) | 9 (34.6%) | 13 (19.1%) | 14 (25.5%) |  |
| **BUN, mg/dL** | 14.15±10.87 | 19.97±16.78 | 14.67±9.81 | 13.61±10.26 | 12.52±8.94 | 0.075 |
| **ALT, U/L** | 201.07±201.71 | 304.53±298.53 | 207.54±237.24 | 178.72±169.17 | 189.89±173.26 | 0.109 |
| **Sodium, mmol/L** | 138.91±7.38 | 138.73±9.85 | 139.05±9.06 | 138.97±6.46 | 138.84±6.80 | 0.999 |
| **Potassium, mmol/L** | 4.02±0.85 | 4.15±0.84 | 4.16±0.86 | 3.90±0.64 | 4.06±1.06 | 0.431 |
| **Calcium, mmol/L** | 1.37±0.53 | 1.30±0.52 | 1.37±0.51 | 1.37±0.53 | 1.39±0.55 | 0.947 |
| **Hemoglobin, g/dL** | 98.55±30.83 | 93.32±24.59 | 100.58±38.15 | 98.03±28.86 | 100.05±31.87 | 0.849 |
| **Glucose, mg/dL** | 9.32±7.20 | 8.84±4.09 | 9.49±3.48 | 9.69±10.26 | 8.94±4.26 | 0.935 |
| **ICU length of stay (day)** | 17.81±14.64 | 13.53±11.47 | 23.96±17.44 | 18.90±15.56 | 15.02±11.95 | 0.034 |
| **Myocardial infarction, n (%)** |  |  |  |  |  | 0.318 |
| Yes | 11 (6.5%) | 3 (15.8%) | 2 (7.7%) | 4 (5.9%) | 2 (3.6%) |  |
| No | 157 (93.5%) | 16 (84.2%) | 24 (92.3%) | 64 (94.1%) | 53 (96.4%) |  |
| **Congestive heart failure, n (%)** |  |  |  |  |  | 0.281 |
| Yes | 34 (20.2%) | 7 (36.8%) | 5 (19.2%) | 13 (19.1%) | 9 (16.4%) |  |
| No | 134 (79.8%) | 12 (63.2%) | 21 (80.8%) | 55 (80.9%) | 46 (83.6%) |  |
| **Cerebrovascular disease, n (%)** |  |  |  |  |  | 0.752 |
| Yes | 56 (33.3%) | 8 (42.1%) | 7 (26.9%) | 22 (32.4%) | 19 (34.5%) |  |
| No | 112 (66.7%) | 11 (57.9%) | 19 (73.1%) | 46 (67.6%) | 36 (65.5%) |  |
| **Chronic pulmonary disease, n (%)** |  |  |  |  |  | 0.216 |
| Yes | 22 (13.1%) | 2 (10.5%) | 1 (3.8%) | 13 (19.1%) | 6 (10.9%) |  |
| No | 146 (86.9%) | 17 (89.5%) | 25 (96.2%) | 55 (80.9%) | 49 (89.1%) |  |
| **Diabetes, n (%)** |  |  |  |  |  | 0.615 |
| Yes | 68 (40.5%) | 8 (42.1%) | 13 (50.0%) | 24 (35.3%) | 23 (41.8%) |  |
| No | 100 (59.5%) | 11 (57.9%) | 13 (50.0%) | 44 (64.7%) | 32 (58.2%) |  |
| **Renal disease, n (%)** |  |  |  |  |  | 0.576 |
| Yes | 84 (50.0%) | 12 (63.2%) | 14 (53.8%) | 33 (48.5%) | 25 (45.5%) |  |
| No | 84 (50.0%) | 7 (36.8%) | 12 (46.2%) | 35 (51.5%) | 30 (54.5%) |  |
| **Cancer, n (%)** |  |  |  |  |  | 0.914 |
| Yes | 43 (25.6%) | 5 (26.3%) | 8 (30.8%) | 16 (23.5%) | 14 (25.5%) |  |
| No | 125 (74.4%) | 14 (73.7%) | 18 (69.2%) | 52 (76.5%) | 41 (74.5%) |  |
| **Vasopressor use, n (%)** |  |  |  |  |  | 0.881 |
| Yes | 5 (3.0%) | 1 (5.3%) | 1 (3.8%) | 2 (2.9%) | 1 (1.8%) |  |
| No | 163 (97.0%) | 18 (94.7%) | 25 (96.2%) | 66 (97.1%) | 54 (98.2%) |  |
| **ICU mortality, n (%)** |  |  |  |  |  | 0.799 |
| Yes | 30 (17.9%) | 3 (15.8%) | 3 (11.5%) | 13 (19.1%) | 11 (20.0%) |  |
| No | 138 (82.1%) | 16 (84.2%) | 23 (88.5%) | 55 (80.9%) | 44 (80.0%) |  |
| **28-day mortality, n (%)** |  |  |  |  |  | 0.002 |
| Yes | 63 (37.5%) | 6 (31.6%) | 7 (26.9%) | 18 (26.5%) | 32 (58.2%) |  |
| No | 105 (62.5%) | 13 (68.4%) | 19 (73.1%) | 50 (73.5%) | 23 (41.8%) |  |
| **90-day mortality, n (%)** |  |  |  |  |  | 0.015 |
| Yes | 82 (48.8%) | 11 (57.9%) | 12 (46.2%) | 24 (35.3%) | 35 (63.6%) |  |
| No | 86 (51.2%) | 8 (42.1%) | 14 (53.8%) | 44 (64.7%) | 20 (36.4%) |  |
|  |  |  |  |  |  |  |

**Supplementary Table 3. Comparison of baseline characteristics of developmental and validation cohorts**

| **Variable** | **Overall (n=1327)** | **Developmental cohort**  **(n=1159)** | **Validation cohort (n=168)** | **P value** |
| --- | --- | --- | --- | --- |
| Age, years | 67.3 [55.2, 77.2] | 67.5 [55.6, 78.1] | 66.0 [54.0, 73.0] | 0.030 |
| BUN, mg/dL | 26.0 [17.0, 43.0] | 25.0 [16.0, 42.0] | 29.6 [20.4, 52.9] | 0.004 |
| ALT, U/L | 44.0 [23.0, 99.2] | 36.5 [21.0, 76.2] | 116 [70.8, 256] | <0.001 |
| Sodium, mmol/L | 138 [135, 141] | 138 [135, 141] | 138 [135, 142] | 0.503 |
| Potassium, mmol/L | 4.20 [3.73, 4.80] | 4.30 [3.80, 4.90] | 3.90 [3.50, 4.30] | <0.001 |
| Hemoglobin, g/dL | 10.7 [8.70, 12.8] | 10.8 [8.90, 12.8] | 9.30 [7.70, 11.9] | <0.001 |
| Glucose, mg/dL | 143 [113, 188] | 143 [113, 186] | 146 [109, 200] | 0.966 |
| Hospital LOS, days | 13.5 [7.94, 22.8] | 13.5 [7.96, 22.6] | 13.5 [7.00, 24.0] | 0.901 |
| Male | 825 (62.2%) | 700 (60.4%) | 125 (74.4%) | <0.001 |
| Myocardial infarction | 269 (20.3%) | 258 (22.3%) | 11 (6.5%) | <0.001 |
| Congestive heart failure | 452 (34.1%) | 418 (36.1%) | 34 (20.2%) | <0.001 |
| Cerebrovascular disease | 209 (15.7%) | 153 (13.2%) | 56 (33.3%) | <0.001 |
| Chronic pulmonary disease | 333 (25.1%) | 311 (26.8%) | 22 (13.1%) | <0.001 |
| Diabetes | 436 (32.9%) | 380 (32.8%) | 56 (33.3%) | 0.888 |
| Renal disease | 369 (27.8%) | 285 (24.6%) | 84 (50.0%) | <0.001 |
| Cancer | 200 (15.1%) | 157 (13.5%) | 43 (25.6%) | <0.001 |
| ICU mortality | 263 (19.8%) | 233 (20.1%) | 30 (17.9%) | 0.495 |
| 28-day mortality | 385 (29.0%) | 322 (27.8%) | 63 (37.5%) | 0.009 |
| 90-day mortality | 480 (36.2%) | 398 (34.3%) | 82 (48.8%) | <0.001 |
| Vasopressor use | 720 (54.3%) | 715 (61.7%) | 5 (3.0%) | <0.001 |
